# Thermoresponsive Copolymer Nanovectors Improve the Bioavailability of Retrograde Inhibitors in the Treatment of *Leishmania* Infections

**DOI:** 10.3389/fcimb.2021.702676

**Published:** 2021-08-19

**Authors:** Evan Craig, Anna Calarco, Raffaele Conte, Veronica Ambrogi, Giovanna Gomez d’Ayala, Philip Alabi, Jason K. Sello, Pierfrancesco Cerruti, Peter E. Kima

**Affiliations:** ^1^Department of Microbiology and Cell Science, University of Florida, Gainesville, FL, United States; ^2^Research Institute on Terrestrial Ecosystems (IRET-CNR), Napoli, Italy; ^3^Department of Chemical, Materials and Production Engineering (DICMaPI) – University of Naples Federico II, Napoli, Italy; ^4^Institute for Polymers, Composites and Biomaterials (IPCB-CNR), Pozzuoli, Italy; ^5^Department of Pharmaceutical Chemistry, University of California, San Francisco, San Francisco, CA, United States

**Keywords:** *Leishmania*, Retro-2, retrograde inhibitors, thermoresponsive polymers, nanoaggregates, encapsulation, bioavailability, drug release

## Abstract

Clinical manifestations of leishmaniasis range from self-healing, cutaneous lesions to fatal infections of the viscera. With no preventative *Leishmania* vaccine available, the frontline option against leishmaniasis is chemotherapy. Unfortunately, currently available anti-*Leishmania* drugs face several obstacles, including toxicity that limits dosing and emergent drug resistant strains in endemic regions. It is, therefore, imperative that more effective drug formulations with decreased toxicity profiles are developed. Previous studies had shown that 2-(((5-Methyl-2-thienyl)methylene)amino)-N-phenylbenzamide (also called Retro-2) has efficacy against *Leishmania* infections. Structure–activity relationship (SAR) analogs of Retro-2, using the dihydroquinazolinone (DHQZ) base structure, were subsequently described that are more efficacious than Retro-2. However, considering the hydrophobic nature of these compounds that limits their solubility and uptake, the current studies were initiated to determine whether the solubility of Retro-2 and its SAR analogs could be enhanced through encapsulation in amphiphilic polymer nanoparticles. We evaluated encapsulation of these compounds in the amphiphilic, thermoresponsive oligo(ethylene glycol) methacrylate-*co*-pentafluorostyrene (PFG30) copolymer that forms nanoparticle aggregates upon heating past temperatures of 30°C. The hydrophobic tracer, coumarin 6, was used to evaluate uptake of a hydrophobic molecule into PFG30 aggregates. Mass spectrometry analysis showed considerably greater delivery of encapsulated DHQZ analogs into infected cells and more rapid shrinkage of *L. amazonensis* communal vacuoles. Moreover, encapsulation in PFG30 augmented the efficacy of Retro-2 and its SAR analogs to clear both *L. amazonensis* and *L. donovani* infections. These studies demonstrate that encapsulation of compounds in PFG30 is a viable approach to dramatically increase bioavailability and efficacy of anti-*Leishmania* compounds.

## Introduction

*Leishmania* are flagellated, obligate, protozoan parasites in the Trypanosomatidae family. They are the causative agents of leishmaniases that manifest as three major clinical entities: cutaneous, mucocutaneous, and visceral infection. The prevalence of leishmaniasis is estimated at 12 to 15 million cases with 350 million people at risk worldwide. Annually, 1.5 to 2 million new leishmaniasis cases occur with approximately 70,000 deaths. In 2016, the World Health Organization (WHO) reported that of 200 countries, 87 (44%) and 75 (38%) countries were classified as endemic for cutaneous leishmaniasis (CL) or visceral leishmaniasis (VL), respectively (Arenas et al., 2017; World Health Organization, 2018; Inceboz, 2019). While CL cases are often self-limiting, VL cases are lethal requiring curative treatment with anti-leishmanial drugs. The WHO has classified leishmaniasis as a neglected disease. The few available drugs have important shortcomings, including high cost, toxicity, and the emergence of resistance strains. Control of VL has been further complicated in regions, including sub-Saharan Africa, that have high rates of human immunodeficiency virus (HIV) infection. HIV-VL-coinfection significantly increases the risk of development of VL as well as decreasing the efficacy of therapeutic responses (Alvar et al., 2008). As there is currently no clinically approved *Leishmania* vaccine, the discovery of new anti-*Leishmania* compounds or strategies to improve current treatments is sorely needed.

A few studies have reported on the antileishmanial efficacy of Retro-2, a small, organic, retrograde pathway inhibitor. Retro-2 was initially identified using high-throughput screens of a chemical library to identify compounds that could limit the toxicity of *Shiga* toxin [*Shiga*-like toxin 1 (SLT1) and 2 (SLT2)], a toxin from enterohemorrhagic *Escherichia coli* or *Shigella dysenteriae*, as well as the plant toxin, ricin. Retro-2 appeared to function through prevention of retrograde transport of these toxins (Stechmann et al., 2010). The efficacy of Retro-2 on *Leishmania* infections was subsequently demonstrated (Canton and Kima, 2012). Craig et al. and other reports later showed that the structural–activity relationship (SAR) analogs, DHQZ 36 and DHQZ 36.1, were more efficacious against both *L. amazonensis* and *L. donovani* infections (Canton and Kima, 2012; Craig et al., 2017; Gupta et al., 2017). While the precise mechanism of action of Retro-2 and its SAR analogs in *Leishmania* infections is not known, its application was shown to result in the shrinkage of the communal *Leishmania* parasitophorous vacuoles (LPVs) that harbor *L. pifanoi* or *L. amazonensis* amastigotes. LPVs had been demonstrated to be hybrid compartments that in addition to their extensive interactions with the endocytic pathway, they also displayed secretory pathway molecules (Canton et al., 2012). Acquisition of secretory pathway molecules was shown to be dependent on the actions of syntaxin 5 (Stx5), a SNARE that participates in vesicle transport and fusion at several points in the secretory pathway (Canton and Kima, 2012; Canton et al., 2012; Arango Duque et al., 2019). Loss of Stx5 function through the expression of a dominant-negative mutant or by siRNA silencing of Stx5 or treatment with Retro-2 resulted in decreased localization of Stx5 to the LPV membrane, as well as a significant decrease in vacuole size and parasite burden (Ndjamen et al., 2010; Canton and Kima, 2012; Canton et al., 2012). Unlike Retro-2, the SAR DHQZ analogs, DHQZ 36 and DHQZ 36.1, are directly *Leishmania*-cidal (Craig et al., 2017).

While improvements in efficacy through SAR analog development are encouraging, Retro-2 and the SAR analogs are limited by their hydrophobic nature. Retro-2 solubility is estimated at <0.4 mg/ml in H_2_O (Carney et al., 2014; Gandhi et al., 2019). It, therefore, requires the use of an organic solvent, such as dimethyl sulfoxide (DMSO), for solubilization and uptake by mammalian cells. One method to significantly improve solubility of hydrophobic compounds while also ensuring protection against degradation is the encapsulation of the drug within nanoparticles. The latter may be made of inorganic or organic, inert, biodegradable materials that form a capsule-like structure around the compound. The benefits of encapsulation include increased solubility and bioavailability, protection against degradation, and the potential for modified release kinetics or targeting to specific sites in the body, thereby reducing toxicity of the compound of interest (Banik et al., 2016; Barouti et al., 2017). This increase in bioavailability while limiting toxicity has resulted in improved safety profiles and efficacy of a multitude of anti-*Leishmania* compounds including liposomal AmB that is currently approved for clinical use (Croft and Coombs, 2003; Nafari et al., 2020). Thus, it is likely that Retro-2 and SAR DHQZ analogs would greatly benefit from nanoparticle encapsulation to improve efficacy of these compounds through improved solubility and delivery.

In this regard, amphiphilic, thermoresponsive polymers are suitable as encapsulation vectors for hydrophobic SAR analogs. They are soluble in organic solvents, while their solubility in water dramatically depends on the temperature. In particular, below a critical temperature (defined as low critical solution temperature, LCST), they form water-soluble nanosized micelles, which are able to encapsulate hydrophobic guest molecules in their hydrophobic core (Almeida et al., 2012). When the temperature exceeds the LCST, hydrogen-bonding interactions between hydrophilic micelle shell and water molecules weaken, and those between hydrophobic moieties become dominant leading to a collapse of polymer chains. The resulting intermicellar aggregates can aptly be used as intracellular drug delivery vectors (Sponchioni et al., 2019).

Recently, it was shown that an amphiphilic statistical copolymer based on oligo(ethylene glycol) methacrylate (OEGMA, *M*_n_ = 300 g/mol) and 30%mol pentafluorostyrene (PFS) (known as PFG30) forms micelles of about 10 nm at room temperature, which self-assemble into 200-nm-sized intermicellar nanoaggregates at temperatures greater than 30°C (Zuppardi et al., 2017). Another study showed that PFG copolymers of different molecular weights were able to efficiently encapsulate and remove a hydrophobic dye, Nile Red, from aqueous solutions by up to 90% removal (Zuppardi et al., 2020). Based on these results, this study evaluates the potential application of PFG30 for the delivery of Retro-2 and its SAR analogs, DHQZ 36 and DHQZ 36.1, to *Leishmania*-infected cells.

## Materials and Methods

### Cell Lines

RAW264.7 murine macrophages were obtained from ATCC (ATCC TIB-71, ATCC, USA) and maintained in Dulbecco’s Modified Eagle’s Medium (DMEM) supplemented with 10% fetal bovine serum (FBS) and 100 μg/ml streptomycin-penicillin. They were cultured in a humidified atmosphere at 37°C with 5% CO_2_.

### Parasites

Promastigotes of *L. amazonensis* strain RAT/BA/74/LV78 (LV78) obtained from Dr. Lynn Soong (UTMB, TX) were maintained at 26°C in Schneider’s Drosophila medium supplemented with 10% heat-inactivated FBS and 10 μg/ml Gentamycin. Promastigotes of *L. donovani* [MHOM/SD/62/1S-C12D (SD)] obtained from Dr. Hira L. Nakhasi laboratory (FDA, MD) was maintained at 26°C in M199 medium supplemented with 10% heat-inactivated FBS and 1% penicillin/streptomycin.

### Materials Preparation and Stock Solutions

Retro-2, DHQZ 36, and DHQZ 36.1 were synthesized by Dr. Jason Sello at Brown University as described previously (Carney et al., 2014; Craig et al., 2017). Stock solutions were prepared in DMSO at concentrations of 20 or 30 mM. Retro-2 was also purchased from Sigma-Aldrich (CAS: 1429192-00-6) and prepared in DMSO, as well as at a concentration of 20 mM. Miltefosine was purchased from Sigma-Aldridge (catalog m5571), and a stock solution was prepared in filtered NanoPure diH_2_O at a concentration of 10 mM. Coumarin 6 (C6) was purchased from Sigma-Aldrich (catalog 546283) and a stock solution was made in DMSO at a 5.3-mM solution. The tetrazolium salt MTT [3-(4,5-dimethylthiazol-2-yl)-2,5-diphenyl tetrazolium bromide] Cell Viability Assay Kit was purchased from Biotium (Fremont, CA).

PFG30 was prepared *via* radical polymerization under nitrogen in DMF as described previously (Zuppardi et al., 2017). PFG30 was dried *via* nitrogen until weight remained constant into a gel-like solid and stored at 4°C. Stock solutions were prepared in sterile-filtered, NanoPure diH_2_O at concentrations of 10 mg/ml *via* gentle mixing in an Eppendorf tube at room temperature overnight. The 10-mg/ml stocks were then stored at 4°C prior to mixing with drugs or C6.

### Modeling Drug Uptake Into PFG30 With C6

C6 solutions were prepared at concentrations between 0.5 and 50 μM in filter-sterilized, NanoPure diH_2_O or incomplete DMEM. C6-loaded PFG30 aggregates were prepared by mixing volumes of a 5.3-mM C6 in DMSO stock solution with a 10-mg/ml PFG30 stock (at a 1:10 w/w C6:polymer ratio) to produce a 20- or 50-μM working PFG30 encapsulated C6 stock solution. This mixture was cooled at 4°C to allow solubilization of any PFG30 precipitate due to DMSO addition, and then heated at 37°C for 5 min to induce polymer self-assembly and C6 encapsulation. Additional warm (37°C) diH_2_O or incomplete DMEM was added for a final volume of 1 ml at a C6 concentration of 20 or 50 μM, respectively. This solution was then serially diluted in warm diH_2_O or incomplete DMEM to produce lower C6-loaded PFG30 aggregate concentrations as described in the text.

For fluorescence or NanoSight experiments, additional C6-free vehicle controls were prepared with DMSO with or without PFG30 encapsulation procedures to account for any signal due to clouding of the solution by polymer or DMSO alone. Both C6 and PFG30-C6 samples had their respective vehicle control fluorescence readings subtracted out to control for any vehicle signal.

To determine if a significant proportion of C6 was not encapsulated, centrifugation experiments were performed alongside C6-loaded PFG30 aggregate samples in fluorescence experiments. Samples for centrifugation were made as described above with aggregates prepared at a 1:10 w/w ratio in filter-sterilized diH_2_O at 37°C. Prior to serial dilution, the stock PFG30:C6 solution made at 50 μM was centrifuged at 10,000*g* in a microcentrifuge for 3 min at 37°C to produce a pellet. Supernatant was then collected from the pellet to remove any free C6 unassociated with the PFG30 pellet. The pellet was then resolubilized in an equal part of diH_2_O and chilled with gentle mixing. This solution was then reheated at 37°C for 5 min to encapsulate the remaining C6, and this solution and the supernatant were diluted in warm diH_2_O for a final nominal concentration of 50 μM. Both sample pellets and the respective supernatants were then diluted to a nominal concentration of 5 μM for fluorescence measurements and compared with the non-centrifuged 5 μM PFG30-C6 sample to estimate the efficiency of the PFG30 encapsulation procedure.

Both sets of samples were then read on a BioTek Cytation™ 5 microplate reader with excitation and emission wavelengths set to 420 and 500 nm, respectively. Vehicle controls with equal portions of DMSO and PFG30 were used to correct for background signal caused through clouding of PFG30 polymers. These values were subtracted out of their respective C6 or PFG30-C6 measurements. Statistically significant changes in fluorescence were calculated through way ANOVA with multiple comparisons using the Holm-Šídák two method.

### PFG30 Encapsulation Procedure

DHQZ analog-loaded PFG30 nanoparticles were prepared by mixing 5 µl of a 20-mM Retro-2, DHQZ 36 or DHQZ 36.1 stock with 32 to 45 µl of a 10-mg/ml PFG30 stock (at a 1:10 w/w drug:polymer ratio). This mixture was cooled at 4°C to remove any PFG30 precipitate and then heated at 37°C for 5 min to induce polymer self-assembly and drug encapsulation. This solution was then raised to a 1-ml volume with warm, complete DMEM to produce a 100-μM DHQZ drug solution loaded into PFG30 aggregates.

When noted, additional PFG30 aggregates housing Retro-2 or miltefosine were sedimented down as described above to pellet PFG30 capsules, and the supernatant was removed by pipette. The pellet was then resolubilized in an equivalent volume of complete DMEM as described above to make a nominal 50 or 10 µM working solution of Retro-2 or miltefosine, respectively. These centrifuged samples were run in tandem as described below with non-centrifuged samples for comparison of PFG30 loading efficiency between the hydrophobic compound, Retro-2, and the hydrophilic compound, miltefosine.

### Nanoparticle Size Characterization and Tracking Analysis

The self-assembly behavior of PFG30 was investigated by dynamic light scattering (DLS), performed with a Malvern Zetasizer Nano ZS instrument (Cambridge, UK) equipped with a 4-mV HeNe laser operating at λ = 633 nm, with a measurement angle of 173°. The measurements were carried out at 20°C and 37°C on a solution made up of 3.4 µl of a 30-mM Retro-2 stock in DMSO, 32 µl of a 10-mg/ml PFG30 stock, and 964.6 ml incomplete DMEM.

To determine if C6 was taken up into the intermicellar aggregates of PFG30, we used NanoSight to visualize and measure PFG30 particles with or without C6. DMSO vehicle or C6 were encapsulated in PFG30 aggregates as described above and diluted in incomplete DMEM without supplementation of 10% FBS because of the high amounts of particles from FBS. After preparation of samples, a dilution was prepared at 1:50 and then run for Nanoparticle Tracking Analysis (NTA, Malvern NanoSight NS300) at 37°C for particle size and concentration at 60 s per replicate. A second 1:50 dilution was then prepared and re-measured with a 500 nm green filter to measure the fraction of particles positive for containing C6. At least two replicates were run in these experiments.

### Immunofluorescence Assay for Drug Susceptibility

RAW264.7 macrophages were plated in 100-mm dishes over glass coverslips at 1 × 10^6^ cells/dish. After 24 h incubation, cells were infected with 96 h stationary phase *L. amazonensis* or 96 h PNA-selected, metacyclic *L. donovani* promastigotes at a 20:1 MOI for 4 h prior to two phosphate-buffered saline (PBS) washes to remove external parasites. Infected macrophages were incubated at 34°C or 37°C, respectively, with 5% CO_2_.

Infected cells were treated with free DHQZ analogs or with PFG30 encapsulated Retro-2, DHQZ 36, or DHQZ 36.1 at a 1:10 w/w (drug:PFG30) ratio as described above. When noted, some samples were centrifuged and supernatant removed as described above prior to treatment. Treatments were performed for 48 h at concentrations ranging from 100 nM to 100 μM. Miltefosine, with or without PFG30 encapsulation, was used as a positive control for parasite clearance from concentrations ranging from 100 nM to 15 μM. Cells were incubated with free drugs or PFG30 encapsulated drugs at 34°C or 37°C for *L. amazonensis* or *L. donovani* infections, respectively, for 48 h. Coverslips were fixed in 4% paraformaldehyde (PFA) in PBS for 1 h prior to storage at 4°C in PBS. Coverslips were then processed in immunofluorescence assays (IFAs) to visualize the distribution of LAMP-1 as described previously (Craig et al., 2017). IFAs were visualized and captured using a QImaging Retiga 1300C cooled CCD camera mounted on an Olympus BX50 microscope equipped with automated filters with 100× NA 1.30 oil-immersion objective. Scored LPVs were delimited by LAMP-1 reactivity and contained at least one parasite nucleus visualized by 4′,6-diamidino-2-phenylindole (DAPI) stain. The percentage of infected macrophages and the average number of parasites was determined by counting at least 100 macrophages per coverslip. Counts were done in duplicate over at least three experiments and EC_50_ values were determined in GraphPad Prism 8 using a four-parameter dose-response best-fit curve line. Statistical significance between treated infected cells, and the control was measured using a one-way ANOVA in GraphPad Prism 8, and multiple comparisons were measured for statistical significance using the Holm-Šídák method.

### Centrifugation Assay for the Estimation of Drug Uptake by PFG30 Aggregates

As described above, PFG30 encapsulated Retro-2 or miltefosine were prepared at 100 μM or 10 μM concentrations, respectively, in warm, complete DMEM. A second set of encapsulated Retro-2 or miltefosine was subjected to centrifugation to form a pellet prior to dilution in DMEM, and the supernatant was discarded. This mixture was re-encapsulated as described above and diluted in warm, complete DMEM prior to treatment of *L. amazonensis*-infected macrophages in tandem with non-centrifuged samples for comparison.

RAW264.7 macrophages were plated and infected with *L. amazonensis* at a 20:1 MOI as described previously. Coverslips were then treated in duplicate with Retro-2 or Miltefosine alone, PFG30-encapsulated Retro-2 or Miltefosine, or centrifugation products of PFG30-encapsulated Retro-2 or Miltefosine for 48 h at 34°C with 5% CO_2_. Cells were then fixed with 2% PFA in PBS and IFA stained for LAMP-1 and DAPI for estimation of infection rates as described above. Statistical significance was measured by one-way ANOVA and the Holm-Šídák method for multiple comparisons.

### Quantitative Uptake of C6-Loaded PFG30 Aggregates Into RAW264.7 Macrophages

To assess the cellular uptake of PFG30 aggregates, RAW264.7 cells were seeded on round, glass coverslips at a density of 4.5 × 10^4^ cells in 24-well plates. On the second day, each well was incubated at 37°C for 0, 30, 60, and 180 min, with 500 ml of a solution made up of 1.7 µl of a 30-mM C6 stock in DMSO, 16 µL of a 10-mg/ml PFG30 stock, and 482.3 ml incomplete DMEM.

Post-treatment at the desired time points, cells were washed twice with PBS and fixed (3.7% formaldehyde in PBS) for 15 min at room temperature. Coverslips were mounted onto microscope slides using ProLong Gold Antifade reagent with DAPI (Thermo Fisher Scientific, Milan, Italy).

Cell-associated fluorescence was qualitatively and quantitatively detected by a Cytation 3 Cell Imaging Multi-Mode Reader fluorescence microscope (Biotek) at 480 nm as previously reported (Calarco et al., 2013).

### Estimation of Retro-2 Incorporation Into RAW264.7 Macrophages by Mass Spectrometry

RAW264.7 macrophages were plated in six-well plates at 1 × 10^6^ cells/well overnight and infected with stationary stage *L. amazonensis* promastigotes as described previously. Cells were then treated 24 h-post infection with 5 or 50 μM free Retro-2 or PFG30-encapuslated Retro-2 in warm, complete DMEM. Treatments were performed at 34°C from 4 to 12 h before preparations of cell lysates with RIPA Buffer (10 mM Tris, pH 7.4, 100 mM NaCl, 1 mM EDTA, 1 mM NaF, 20 mM Na_4_P_2_O_7_, 2 mM Na_3_VO_4_, 1% NP-40, 0.5% deoxycholate) supplemented with 1 mM PMSF and protease cocktail inhibitor, purchased from Sigma Aldrich, prior to use. Lysates were sonicated for 15 s prior to centrifugation to remove cellular debris. Supernatants and cell lysates were frozen at −80°C until ready for analysis.

Mass spectrometry analysis of the cell lysates was performed on a Shimadzu Ultra-High-Performance Liquid Chromatography (Nexera XR LC 40), coupled to an MS/MS detector (LCMS 8060, Shimadzu), and controlled by Lab Solution software. The MS/MS was operated in positive electrospray ionization (ESI+), setting the nebulizing gas flow at 3 L/min, heating gas flow 10 L/min, interface temperature 370°C, DL temperature 250°C, heat block temperature 450°C, and drying gas flow 10 L/min. Analyzed fragments of Retro-2 (343.00 > 39.05; 343.00 > 23.15; 343.00 > 284.3) were selected by flow injection analysis mass identification, while quantification was operated after chromatographic separation of acetonitrile treated lysates (1:1 v/v) on a Phenomenex Kinetex polar C18 column (3 × 100 mm, 2.6 μm, Phenomenex, USA) with mobile phase consisting of acetonitrile: water + 0.01% formic acid (70:30, v/v; isocratic). A bar graph with estimated internal Retro-2 concentrations was generated in GraphPad Prism 8 and statistical significance measured *via* two-way ANOVA and the Tukey method for multiple comparisons.

### Measurement of Vacuole Size

LPV size measurements were performed as described previously (Craig et al., 2017). Briefly, RAW264.7 macrophages on coverslips were infected with stationary *L. amazonensis* promastigotes at 10:1 MOI for 4 h after which parasites were washed off and DHQZ analogs, PFG30 encapsulated DHQZ analogs or miltefosine were added for an additional 24 h. Infected macrophages were fixed with 2% PFA in PBS then processed in IFAs to visualize the distribution of LAMP-1. IFAs were visualized and captured using a QImaging Retiga 1300C cooled CCD camera mounted on an Olympus BX50 microscope equipped with automated filters with 100× NA 1.30 oil-immersion objective. Scored LPVs were delimited by LAMP-1 reactivity and contained at least one parasite nucleus visualized by DAPI stain. Vacuole size was determined by measuring vacuole area *via* ImageJ. At least 30 vacuoles were measured per coverslip of which there were three per concentration per biological repeat. Statistical significance between treated infected cells and the control was measured using a one-way ANOVA in GraphPad Prism 8 with the Dunnett test for multiple comparisons.

### Estimation of PFG30 Cytotoxicity

RAW264.7 macrophages were seeded at 1 × 10^4^ cells/well in 96-well plates in complete DMEM at 37°C with 5% CO_2_ for 2 to 4 h to allow adherence, but not replication. Cells were then treated with filter-sterilized, NanoPure diH_2_O vehicle or PFG30 at concentrations ranging from 450 to 1 mg/ml for 48 h prior to MTT assay. After treatment, cells were incubated an additional 4 h with MTT before addition of 10% SDS in PBS with 5 N HCl to dissolve formazan crystals overnight. Absorbances were read at 570 nm with a 630 nm background subtracted out. Cell numbers were estimated based on a standard curve and statistical significance was measured *via* one-way ANOVA with multiple comparisons using the Holm-Šídák method.

## Results

### PFG30 Enhances C6 Solubility Through Efficient Uptake Into Nanoaggregates

Previous studies had shown that Retro-2 and two SAR analogs, DHQZ 36 and DHQZ 36.1 are effective compounds against *L. amazonensis* and *L. donovani* infections (Canton and Kima, 2012; Craig et al., 2017; Gupta et al., 2017). However, these compounds are hydrophobic with low solubility in aqueous solutions. It is, therefore, likely that the efficacy of these compounds could be further enhanced by increasing their solubility through polymer-based encapsulation. Polymer encapsulation has been shown to increase hydrophobic drug solubility, drug delivery, and protection from degradation *in vivo* (Huh and Kwon, 2011; Costa Lima et al., 2012; Nafari et al., 2020). Previously, amphiphilic co-polymers made from OEGMA and 30% PFS were found to be responsive to temperatures greater than 30°C, leading to stable nanoaggregates with a hydrophobic interior (Lutz, 2011; Kwan and Marić, 2016; Welsch and Lyon, 2017). Therefore, it is expected that these nanoparticles may enhance solubility and bioavailability of hydrophobic Retro-2 and SAR analogs.

**Figure 1:**
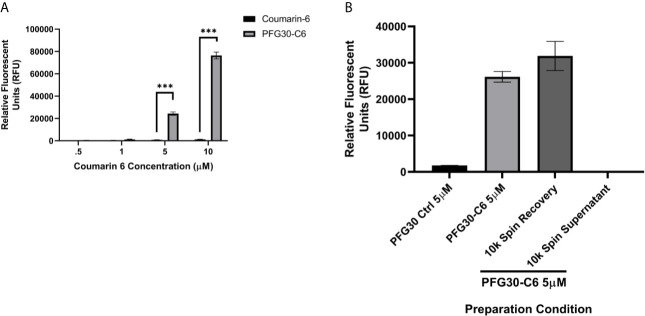
Encapsulation of hydrophobic molecules in PFG30 increases their solubility. **(A)** Increasing concentrations of C6 alone or PFG30 + C6 was measured at 420 nm excitation and 500 nm emission. **(B)** 50 µM PFG30-C6 preparations were centrifuged after which the supernatant fluid and pellets were diluted to 5 µM for measurement of fluorescence at 420/500 nm. Experiments were run in triplicate with at least two technical replicates each. Statistical significance was determined by two-way ANOVA in GraphPad Prism 8 with the Holm-Šídák posthoc test for multiple comparisons (***p-value < 0.001).

In the first series of experiments, the uptake of poorly soluble, hydrophobic molecules into PFG30 thermoresponsive aggregates was assessed. As Retro-2 is not fluorescent and its UV absorption spectrum overlaps with that of PFG30, we evaluated PFG30’s ability to encapsulate and deliver the green laser dye, coumarin 6 (C6), to obtain further insight into its efficiency of encapsulating hydrophobic compounds (Zuppardi et al., 2020). The fluorescence of C6 is low in polar solvents bcause of its poor solubility; however, its fluorescence can be restored by using amphiphilic hosts that have hydrophobic cavities (Banerjee et al., 2016). The solubility of C6 in water with or without PFG30 encapsulation was measured through changes in the fluorescent profile. Fluorescence of 5 and 10 µM C6 in the presence of PFG30 was significantly greater at RFU values of 24,381 and 76,430, respectively, than water-dispersed C6, which barely fluoresced at 810 and 1,319 RFU, respectively (**Figure 1A**). The experiment was stopped at 10 µM because of the values reaching overflow in PFG30 encapsulated C6 samples.

To determine if increased fluorescence of C6 was due to PFG30 encapsulation, we elected to separate free C6 in solution from encapsulated C6 *via* centrifugation. After removal of the supernatant and resuspension of the pellet, there was no significant drop in the fluorescence of the pellet of the 5 µM sample at 31,888 RFU compared to the control 5 µM sample (26,160 RFU) without centrifugation. In addition, the supernatant contained very little C6 with a fluorescent value of only 189 RFU (**Figure 1B**).

Because of the increase in C6 solubility and association with the PFG30 pellet in centrifugation experiments, DLS was used to assess whether PFG30 was able to retain its thermally triggered self-assembly properties in DMEM solution, as well as in the presence of the hydrophobic compound Retro-2. To that end, the size distribution of a PFG30 solution containing Retro-2 in DMEM was measured by DLS at 20°C and 37°C. At 20°C, polymer micelles having average hydrodynamic diameter of 10 nm were detected, with monomodal distribution and low polydispersity. When the temperature was raised to 37°C, the particle size increased up to about 200 nm, due to formation of intermicellar aggregates, demonstrating that Retro-2 did not affect the self-assembly behavior of PFG30 (**Supplemental Figure 1**).

**Figure 2:**
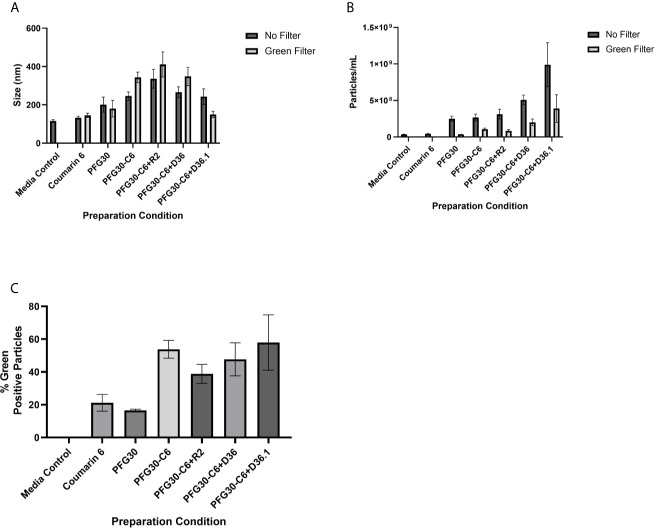
NanoSight measurements of PFG30 encapsulated compounds. The size and number of particles produced after encapsulation in PFG30 were analyzed as follows: C6 alone (20 μM); PFG30 alone; PFG30 + C6 (20 μM); PFG30 + C6 + Retro-2 (50 μM R2); PFG30 + C6 + DHQZ 36 (50 μM); PFG30 + C6 + DHQZ 36.1 (50 μM). Encapsulation mixtures were prepared at 1:50 dilution for NanoSight analysis. **(A)** The size measurements of polymer aggregates with or without the use of a 500 nm green filter for fluorescence. **(B)** The number of particles within the samples with or without the use of a 500 nm green filter for fluorescence. **(C)** Particles observed in samples using a 500-nm green filter were compared with the total particles in the sample observed without the filter to generate a percentage estimation of C6 uptake within PFG30 aggregates. Data were obtained from at least two biological replicates.

To further affirm that the increased solubility of C6 was indeed due to encapsulation of C6 into PFG30, the sizes of PFG30 intermicellar aggregates, with or without C6, were assessed by NanoSight. PFG30 aggregates without C6 were about 200 nm in size. Encapsulation of C6 with PFG30 resulted in increased particle sizes ranging from 200 to 400 nm in size. The incomplete DMEM vehicle included a small population of particles. However, they were distinctly different from PFG30 nanoparticles with sizes less than 100 nm (**Figure 2A**). Furthermore, the addition of PFG30 significantly increased the magnitude of particles compared to incomplete DMEM or incomplete DMEM with free C6 (**Figure 2B**). Using a 500 nm wavelength filter for green fluorescence in the NanoSight, we found that there was a significant increase in the number of green fluorescent particles in PFG30 encapsulated C6 samples as compared with PFG30 or C6 alone. These results suggests that C6 is encapsulated within PFG30 aggregates.

Considering the observations on the encapsulation of C6, the encapsulation of Retro-2 and its SAR derivatives, DHQZ 36 and DHQZ 36.1 were evaluated. 50 μM Retro-2, DHQZ 36 or DHQZ 36.1 mixed with C6 were encapsulated by PFG30 at a 1:10 w/w ratio. A 1:50 dilution in incomplete DMEM was prepared for evaluation by NanoSight. Mixing of C6, DHQZ analogs, and PFG30 resulted in an increase in the size of the particles that were formed (**Figure 2A**). There was also an increase in the number of PFG30 aggregates that were formed (**Figure 2B**). Interestingly, PFG30 particles formed upon addition of DHQZ 36 and DHQZ 36.1, were smaller but more numerous (**Figures 2A, B**). Retro-2 and the two DHQZ analogs mixed with C6 had a similar increase in the concentrations of green-positive particles as compared to PFG30 encapsulation of C6 alone, suggesting that the addition of drug did not affect C6 loading into PFG30 aggregates (**Figure 2C**).

### PFG30 Enhances Efficacy of SAR DHQZ Analogs of Retro-2 Against *L. donovani* and *L. amazonensis* Infections of RAW264.7 Macrophages

We commenced these studies by assessing whether Retro-2 efficacy against intracellular *Leishmania* amastigotes would be improved by encapsulation in PFG30 polymeric nanoparticles.

**Figure 3:**
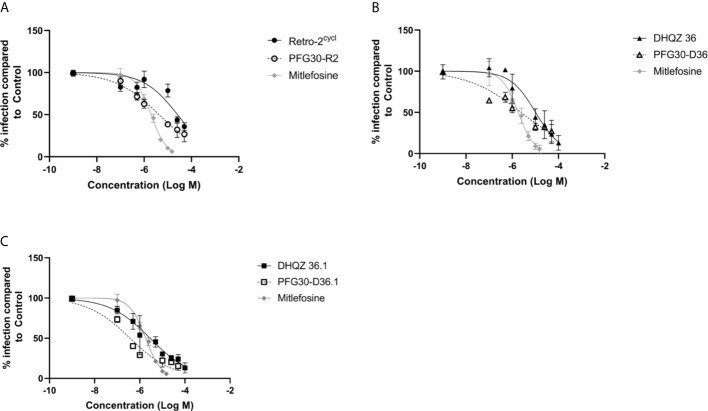
Evaluation of polymer-encapsulated compounds on *L. donovani*-infected RAW264.7 macrophages. *L. donovani*-infected macrophages were treated for 48 h with the following compounds: **(A)** free Retro-2 or PFG30 + Retro-2 (PFG30-R2); **(B)** free DHQZ 36 or PFG30 + DHQZ 36 (PFG30-D36); **(C)** free DHQZ 36.1 or PFG30 + DHQZ 36.1 (PFG30-D36.1) at concentrations ranging from 100 nM to 100 μM. Miltefosine (100 nM to 15 μM) treatment was used as a positive control for parasite clearance. Cells were fixed in 2% PFA in PBS. IFAs were performed for detection of LAMP-1 and cell and parasite nuclei with DAPI. At least 200 cells were scored per coverslip and infection rates were standardized to a vehicle control before EC_50_ estimation. Data were compiled from at least three biological repeats.

RAW264.7 macrophages plated on coverslips in six-well plates were infected with *L. donovani* parasites for 24 h. Thereafter, several concentrations of Retro-2, DHQZ 36, or DHQZ 36.1 encapsulated in PFG30 were added to infections. After an additional 48 h, the infections were fixed by adding 2% PFA. Coverslips with infected cells were then processed by immunofluorescence labeling to detect the contours of the LPV and to visualize parasite nuclei. After enumerating the number of cells harboring intracellular *Leishmania*, the data were plotted (**Figure 3**). PFG30 encapsulation of Retro-2 significantly augmented the EC_50_ value calculated at 4.46 ± 0.94 μM compared to unencapsulated Retro-2 at 22.73 ± 3.44 μM. Due to the increased efficacy observed of Retro-2 encapsulated in PFG30 to clear *L. donovani* infections, we proceeded to test the efficacy of DHQZ 36 and DHQZ 36.1 encapsulated in PFG30. After processing in immunofluorescence assays and enumeration of infected macrophages, we determined that DHQZ 36 encapsulated in PFG30 had an EC_50_ value of 1.07 ± 0.98 μM (**Figure 3B**). Encapsulated DHQZ 36.1 had an EC_50_ value of 0.27 ± 0.09 μM, (**Figure 3C**). **Table 1** shows the EC_50_ of DHQZ 36 and DHQZ 36.1 without encapsulation. The efficacy of DHQZ compounds and encapsulated formulations were run alongside miltefosine. The EC_50_ of miltefosine in these experiments was determined to be 1.96 ± 0.38 μM. As shown in **Table 1**, the EC_50_ values of the PFG30 encapsulated compounds on *L. donovani* infections were much improved and were effective at lower concentrations than miltefosine.

**Table 1:** Estimation of the efficacy of PFG30 encapsulated DHQZ analogs on *L. donovani* infections.

Treatment	EC_50_ (μM)	
	PFG30 (−)	PFG30 (+)
Retro-2	22.73 ± 3.44	4.46 ± 0.94***
DHQZ 36	11.70 ± 0.33	1.07 ± 0.98***
DHQZ 36.1	1.80 ± 0.20	0.27 ± 0.09***
Miltefosine	1.96 ± 0.38	

R2, Retro-2; D36, DHQZ 36; D36.1, DHQZ 36.1.

EC_50_ values were calculated for the plots in **Figure 3** generated in GraphPad Prism 8. Statistical significance was measured between LogEC_50_ values between DHQZ compounds and encapsulated DHQZ compounds though the Extra Sum-of-Squares F test (***p-value < 0.001).

**Figure 4:**
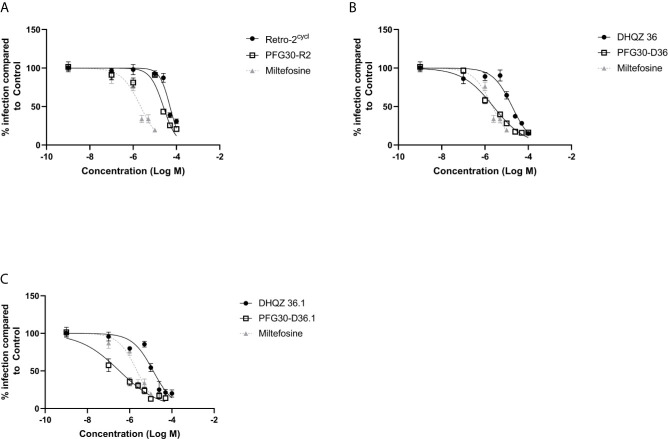
Evaluation of polymer-encapsulated compounds on *L. amazonensis*-infected RAW264.7 macrophages. To *L. amazonensis*-infected RAW264.7 macrophages, the following drug/polymer mixtures were added then incubated for an additional 48 h. **(A)** Retro-2 in DMSO or Retro-2 + PFG30; **(B)** DHQZ 36 in DMSO or DHQZ 36 + PFG30; **(C)** DHQZ 36.1 in DMSO or DHQZ 36.1 + PFG30 at concentrations of 100 nM to 100 μM. Miltefosine (100 nM to 15 μM) treatment served as a positive control for parasite clearance. Cells were fixed in 2% PFA in PBS. IFAs were performed for detection of LAMP-1 and cell and parasite nuclei with DAPI. At least 200 cells were scored per coverslip and infection rates were standardized to a vehicle control before EC_50_ estimation. Data were compiled from at least three biological repeats.

We then proceeded to test the efficacy of the encapsulated compounds just described, on *L. amazonensis* infections. We wanted to determine if using the same methodology for PFG30 encapsulation would improve the efficacy Retro-2 and the DHQZ analogs on *L. amazonensis* infections. The drug killing curves that were generated for the studies on *L. amazonensis* infections are shown in **Figure 4**. From these curves we calculated the EC_50_ for PFG30 encapsulated Retro-2 to be 26.15 ± 2.46 μM; the EC_50_ of encapsulated DHQZ 36 was 2.42 ± 2.12 μM; the EC_50_ of PFG30 encapsulated DHQZ 36.1 was 0.36 ± 0.13 μM. These results are presented in **Table 2** alongside the EC_50_ values of these compounds without encapsulation. **Supplemental Figure 2** and **Table 2** show that encapsulation of miltefosine, using the encapsulation protocol described above, did not result in a significant change in the EC_50_ of miltefosine. The EC_50_ of miltefosine on *L. amazonensis* infections is 2.50 ± 0.37 μM.

**Table 2:** Estimated efficacy on *L. amazonensis* infections of Retro-2 and DHQZ analogs with or without PFG30.

Treatment	EC_50_ (μM)	
	PFG30 (−)	PFG30 (+)
Retro-2	50.29 ± 5.91	26.15 ± 2.46***
DHQZ 36	18.00 ± 2.54	2.42 ± 2.12***
DHQZ 36.1	13.05 ± 0.49	0.36 ± 0.13***
Miltefosine	2.23 ± 0.27	2.11 ± 0.26

EC_50_ values were calculated from fitted-line dose-response curves in **Figure 4** of R2, Retro-2; D36, DHQZ 36; D36.1, DHQZ 36.1 Plots and analysis were performed in GraphPad Prism7. Statistical significance was measured between LogEC_50_ values between Retro-2 and encapsulated Retro-2 though the Extra Sum-of-Squares F test (***p-value < 0.001).

We proceeded to rule out the possibility that the increase in efficacy of the encapsulated compounds was not due to toxicity of PFG30 on RAW264.7 macrophages, which may lead to stress of the parasites during infection. This was determined by using a MTT assay to measure RAW264.7 cell viability with PFG30 treatment over 48 h. In this experiment, we included much higher concentrations of PFG30 along with a filter-sterilized, NanoPure diH_2_O vehicle control. No significant loss in cell viability was observed at 48 h treatments of RAW264.7 macrophages, even at 1 mg/ml concentrations of PFG30 (**Supplemental Figure 3**).

### Retro-2 SAR Analogs Are Efficiently Packaged in PFG30 Aggregates and Delivered Into Macrophages *In Vitro*

**Figure 5:**
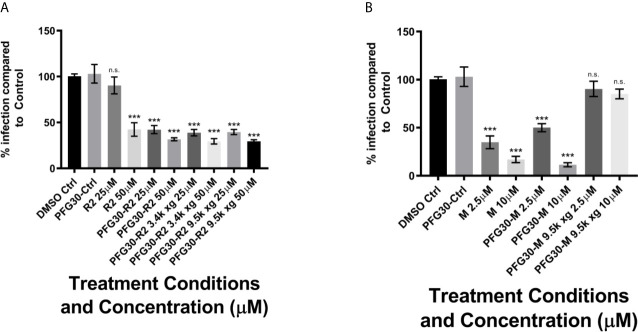
Evaluation of PFG30 encapsulation efficiency of Retro-2 and miltefosine through centrifugation separation of free and encapsulated drug. *L. amazonensis-*infected RAW264.7 macrophages were treated for 48 h with the following preparations: **(A)** Retro-2, PFG30 + Retro-2 or PFG30 + Retro-2 post-centrifugation; **(B)** miltefosine, PFG30 + miltefosine or PFG30 + miltefosine post-centrifugation. Cells were fixed in 2% PFA in PBS. IFA’s were performed for detection of LAMP-1 and cell and parasite nuclei with DAPI. Statistical significance was determined by one-way ANOVA in GraphPad Prism 8 with the Holm-Šídák posthoc test for multiple comparisons (n.s., not statistically significant, ***p-value < 0.001). Data were compiled from three biological experiments with at least two technical replicates.

While PFG30 encapsulation experiments against *L. donovani* and *L. amazonensis* infections indicate improved efficacy, it is uncertain how much DHQZ analogs are retained in the PFG30 polymer capsules and how much drug is excluded. Therefore, we reasoned that due to the physical aggregation of the capsules, these could be centrifuged into a pellet allowing for the removal of the supernatant fluid containing any free drug unassociated with the polymers. Once free drug was removed, the remaining drug associated with the polymer could be tested against *L. amazonensis* infections and potential loss of efficacy determined. We observed that with 10,000*g* centrifugation at 37°C, pelleted Retro-2 encapsulated in PFG30 had no significant loss in efficacy after supernatant was discarded (**Figure 5A**). In contrast, centrifugation of PFG30 encapsulated miltefosine and removal of supernatant from the PFG30 pellet resulted in significant loss of efficacy even at 10 μM (**Figure 5B**), likely because of the hydrophilicity of the compound.

**Figure 6:**
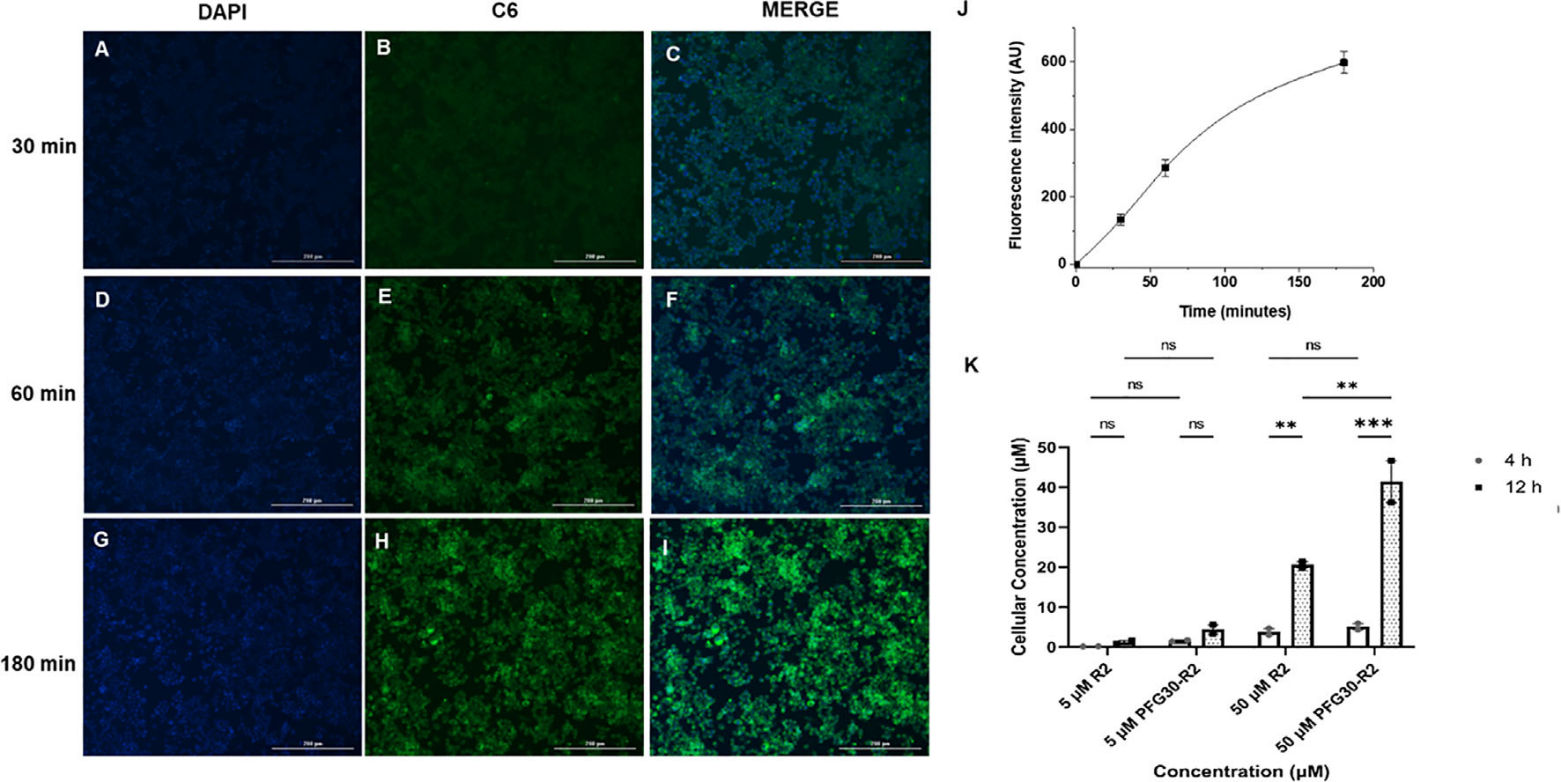
PFG30 encapsulation of Retro-2 (R2) increases uptake of the drug into RAW264.7 macrophages. **(A–I)** Representative microscope images (magnification 10X) of PFG30 + C6 nanoparticle uptake by RAW264.7 macrophages at different incubation time. RAW264.7 macrophages were incubated at 37°C with PFG30 encapsulated C6 at the following time points: **(A–C)** 30 min, **(D–F)** 60 min, and **(G–I)** 180 min. **(J)** Cell-associated fluorescence quantification calculated as a function of time. The curve was generated by performing image analysis (ImageJ software). Results are expressed as the mean of three biological replicates ± standard deviation (n = 3). **(K)** LC/MS quantification of R2 concentration in *L. amazonensis-*infected RAW264.7 macrophages. Cells were treated with R2 or PFG30 encapsulated R2 (PFG30-R2) for 4 or 12 h. At the indicated times, cells were recovered and supernatant was collected. The cell pellets were lysated in RIPA buffer and then sonicated for 15 s twice prior to storage at –80°C. Statistical significance was determined by two-way ANOVA in GraphPad Prism 8 with the Tukey posthoc test for multiple comparisons (ns, not statistically significant where p-value > 0.05, **p-value < 0.01, ***p-value < 0.001). Data were compiled from two biological replicates.

Because of the results with C6 solubility and centrifugation experiments, it is likely that Retro-2 SAR analogs are also solubilized and packaged into PFG30 nanoaggregates. Therefore, PFG30 encapsulation and uptake could facilitate Retro-2 entry into infected cells leading to the observed increase in efficacy. To first estimate PFG30 uptake into cells, RAW264.7 macrophages were treated with PFG30 encapsulated C6 up to 180 min prior to fixation. These cells were then stained with DAPI and visualized *via* fluorescence microscopy to assess PFG30 uptake by macrophages. The intracellular C6-associated fluorescence was visualized as early as 30 min post-treatment and increased gradually until 180 min (**Figures 6A–I**). The increasing amount of C6 uptake into cells was confirmed by fluorescence quantification (**Figure 6J**). Additional experiments related to C6-loaded PFG30 uptake were performed on mouse fibroblast cell line (L929). The result clearly demonstrated that PFG30 nanoparticles efficiently enter the cells, suggesting that the PFG30 cellular uptake is not related to the phagocytic nature of the cell (**Supplemental Figure S4**).

Based on these results, the concentration of Retro-2 uptaken by cells after PFG30 encapsulation, was then evaluated by LC/MS (**Figure 6K**). Infected cells were treated with various amounts of PFG30 encapsulated Retro-2 for 4 and 12 h before lysis by the addition of RIPA buffer and sonication. Early timepoints were used to limit potential drug loss from drug turnover within cellular compartments, as this compound is relatively stable in acidic conditions (Gandhi et al., 2019). Mass spectrometry analysis of the cell lysates found that levels of Retro-2 treatments of 5 and 50 µM in macrophage lysates were detectable at 4 h post-treatment with cellular uptake at 0.14 ± 0.04 µM and 3.83 ± 1.15 µM, respectively. These concentrations increased over time at 12 h post-treatment to 1.18 ± 0.59 µM in the 5-µM treated sample and 20.62 ± 1.11 µM in the 50-µM treated sample. Drug levels in cells were found to be elevated with PFG30 encapsulation at all time points. At 4 h, PFG30-Retro-2 treatments of 5 and 50 µM resulted in intracellular Retro-2 levels at 1.55 ± 0.28 µM and 5.1 ± 1.13 µM, respectively. This effect was more pronounced at 12 h post-treatment with intracellular Retro-2 concentrations of 4.43 ± 1.59 µM and 41.45 ± 7.42 µM in the 5 and 50 µM PFG30-Retro-2 treated samples, respectively (**Figure 6K**).

### PFG30 Aggregates Improve Effects of DHQZ Analogs on LPV Maturation Through Prevention of Vacuole Size Increases During *L. amazonensis* Infection

**Figure 7:**
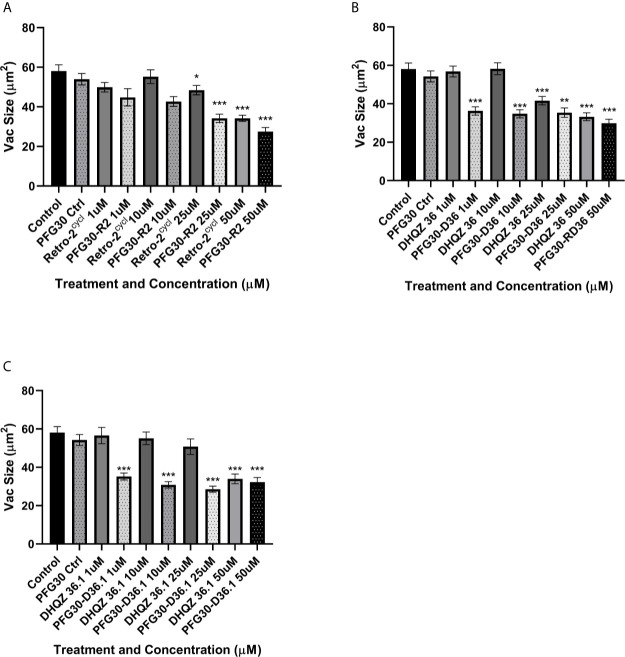
Vacuole size measurements of drug treated *L. amazonensis* LPVs. RAW264.7 macrophages were infected for 24 h after which they were treated Retro-2 or DHQZ analogs, with or without encapsulation in PFG30. **(A)** Treatment with Retro-2 (0–50 µM) with or without PFG30 encapsulation. **(B)** Treatment with DHQZ 36 (0–50 µM) with or without encapsulation. **(C)** Treatment DHQZ 36.1 (0–50 µM) with or without PFG30 encapsulation. Data were compiled from three experiments. At least 30 vacuoles were measured per treatment *via* one-way ANOVA with the Šídák correction for multiple comparisons. (*p-value < 0.05, **p-value < 0.01, ***p-value < 0.001).

Previously, we showed that treatment with Retro-2 and the DHQZ analogs, DHQZ 36 and DHQZ 36.1, resulted in reduced sizes of maturing *L. amazonensis* LPVs (Craig et al., 2017). Considering those observations, we wanted to determine if the effects on maturing LPVs DHQZ analogs could be enhanced with PFG30 encapsulation. RAW264.7 cells were infected with *L. amazonensis* promastigotes for 4 h prior to treatment with DHQZ analogs, alone or PFG30 encapsulated, for an additional 24 h. We found that PFG30 enhanced the vacuole size reduction results observed previously with drug alone with all three compounds. Free Retro-2 significantly reduced vacuole size in *L. amazonensis*-infected RAW264.7 macrophages at 25 and 50 µM by 16.6% and 41.2%, respectively. PFG30 encapsulated Retro-2 increased this effect with significant loss of vacuole size by 21.2% at 10 µM (**Figure 7A**). DHQZ 36 also showed significant vacuole size reductions at 25 and 50 µM by 28.3% and 42.7%, respectively. PFG30 encapsulation of DHQZ 36 improved this effect significantly at concentrations as low as 1 µM with a size reduction of 33.1% compared with the PFG30-treated control (**Figure 7B**). Free DHQZ 36.1 only showed significant size reduction at 50 µM by 41.5%. However, PFG30 encapsulation of DHQZ 36.1 showed similar results to DHQZ 36 with 1 µM significantly reducing vacuole size by 35.0% compared to the control (**Figure 7C**).

## Conclusions/Discussion

With no preventative vaccine available for leishmaniasis, chemotherapy is the front-line option for treatment of this potentially deadly disease. However, the pool of clinically-available anti-*Leishmania* compounds remains relatively small. One major issue in drug development of new potentially effective compounds is the problem of solubility. It is estimated that approximately 40% of market compounds and up to 90% of pipeline candidate drugs are poorly soluble in water (Kalepu and Nekkanti, 2015). Although repurposing drugs is a viable strategy for discovery of anti-*Leishmania* candidates because of the preceding studies of their bioavailability and toxicity *in vivo*, it would nonetheless be beneficial to utilize the large pool of hydrophobic compounds in the discovery pipeline. One way to overcome the hurdle of water solubility is the encapsulation of drugs in water-soluble, inert, biodegradable nanoparticle capsules to enhance drug solubility (Zhang et al., 2008; Rao and Geckeler, 2011). In this manuscript, we evaluated PFG30 for their capacity to encapsulate and enhance the efficacy of Retro-2 and its DHQZ SAR analogs to clear *L. donovani* and *L. amazonensis* infections of macrophages *in vitro*.

We first decided to explore the efficiency of PFG30 to encapsulate hydrophobic compounds. C6 was selected due to its fluorescent properties and low solubility in aqueous solution. In addition, Zuppardi et al. recently used PFG preparations of different polyfluorostyrene concentrations with a similar dye, Nile Red, to demonstrate efficient removal of the dye from aqueous solution (Zuppardi et al., 2020). When encapsulated by PFG30, C6 solubility was significantly increased in water compared with C6 alone as measured by 420/500 nm excitation and emission fluorescence. This increase in solubility appears to be due to C6 encapsulation into PFG30 aggregates as tested through centrifugation and NanoSight experiments. Despite removal of the supernatant, C6 fluorescence did not significantly change compared to PFG30 encapsulated C6 without centrifugation. While PFG30 did cause some background fluorescent particles likely through light refraction, the magnitude of green fluorescent particles measured on NanoSight were higher in PFG30 encapsulated C6 samples compared to C6 or PFG30 alone. DLS experiments with PFG30 encapsulation of Retro-2 at 20°C and 37°C show that thermosensitive polymer self-assembly was not impeded upon uptake of hydrophobic cargo.

Because of the improved solubility of the hydrophobic molecule, C6, after encapsulation by PFG30, we tested the improved efficacy of encapsulated Retro-2, DHQZ 36, and DHQZ 36.1 against *L. donovani* and *L. amazonensis in vitro* infections of RAW264.7 macrophages. PFG30 encapsulation was found to enhance the efficacy of all three DHQZ compounds with DHQZ 36.1 EC_50_ values in the nanomolar range, surpassing that of miltefosine, which is used clinically. It is likely that these compounds are encapsulated within PFG30 aggregates similar to encapsulation of C6 as centrifugation experiments and sampling of the pellet did not affect the EC_50_ of DHQZ analogs encapsulated in PFG30. This suggests that these compounds are likely enclosed in the hydrophobic portion of the PFG30 aggregate. Conversely, the EC_50_ of miltefosine against *L. amazonensis* was not significantly affected by PFG30 encapsulation, and efficacy was lost upon centrifugation and sampling of the pellet. These results suggest that miltefosine was not efficiently encapsulated within PFG30 aggregates. Likely, because of its hydrophilic nature, miltefosine remained in the aqueous supernatant, which was removed post-centrifugation.

Because of the increase in solubility of C6 and the increased efficacy of Retro-2 DHQZ SAR analogs, we decided to test if this effect was due to improved delivery of Retro-2 by uptake of PFG30 aggregates using LC/MS. Small, but not significant, increases were observed as early as 4 h post-treatment with PFG30 encapsulated Retro-2 compared to free Retro-2. However, this increase was much more pronounced by 12 h post-treatment especially in the 50-µM sample where 82.9% of the encapsulated Retro-2 was associated with the cellular lysate. This result suggests that PFG30 aggregates are efficiently taken up by RAW264.7 macrophages and that encapsulation of Retro-2 is a viable strategy for improving its delivery to infected cells. However, the dynamics of uptake and release of Retro-2 encapsulated in PFG30 aggregates remains unknown. The dramatically increased reduction of LPV size by PFG30 encapsulated DHQZ 36 and DHQZ 36.1 at 24 h post-treatment suggests that a large proportion of the compounds are able to target the host secretory pathway machinery. Additionally, due to the reported direct activity of Retro-2 analogs against *Leishmania*, it is possible that encapsulation increases delivery of Retro-2 directly to amastigotes residing within the LPV (Canton and Kima, 2012; Craig et al., 2017). *Leishmania* LPVs acquire large amounts of LAMP-1-associated membrane which may lead to the direct delivery of endocytic vesicles containing PFG30 encapsulated DHQZ analogs.

This study presents PFG30 as a potential candidate copolymer for biological application as a drug carrier. Further studies should elucidate its uptake and release mechanisms within cells as well as its viability for use in *in vivo* studies. In addition, this study highlights the significant improvement of DHQZ analog efficacy with polymer encapsulation. It is likely that other nanocarrier options would provide similar improvements. As an organic polymer, PFG30 may also be further improved through the addition of targeting mechanisms to increase uptake and cell specificity. These methods may further enhance drug delivery and bioavailability within an *in vivo* model.

## Data Availability Statement

The original contributions presented in the study are publicly available. This data can be found here: https://figshare.com/articles/dataset/Thermoresponsive_copolymer_nanovectors_improve_the_bioavailability_of_retrograde_inhibitors_LC-MS_dataset_xlsx/14553783.

## Author Contributions

EC performed experiments and wrote manuscript: RC, GG performed experiments. VA and AC performed experiments and contributed analysis and writing. PA and JS synthesized most drugs used in study. PC made intellectual contributions to the study and wrote and edited manuscript. PK made intellectual contributions to the study and wrote and edited manuscript and was responsible for the overall execution of the study. All authors contributed to the article and approved the submitted version.

## Conflict of Interest

The authors declare that the research was conducted in the absence of any commercial or financial relationships that could be construed as a potential conflict of interest.

## Publisher’s Note

All claims expressed in this article are solely those of the authors and do not necessarily represent those of their affiliated organizations, or those of the publisher, the editors and the reviewers. Any product that may be evaluated in this article, or claim that may be made by its manufacturer, is not guaranteed or endorsed by the publisher.

## Supplementary Material

Supplementary Figure 1 Size distribution of PFG30 + Retro-2 particles measured by DLS at 20°C and 37°C. The self-assembly behavior of PFG30 was investigated by DLS performed at 20°C and 37°C on a solution containing Retro-2, PFG30, and incomplete DMEM. At 20°C, 10 nm polymer micelles were detected, whereas at 37°C intermicellar aggregates of about 200 nm in size formed, demonstrating that encapsulation of Retro-2 did not affect the self-assembly behavior of PFG30. The reported size distribution curves are averaged from 3 experiments.

Supplementary Figure 2 Evaluation of polymer-encapsulated miltefosine on *L. amazonensis*-infected RAW264.7 macrophages. To *L. amazonensis*-infected RAW264.7 macrophages, free or PFG30 encapsulated miltefosine was added. After 48 h treatment, cells were fixed in 2% PFA in PBS. IFA’s were performed for detection of LAMP-1 and cell and parasite nuclei with DAPI. At least 200 cells were scored per coverslip and infection rates were standardized to a vehicle control before EC_50_ estimation. Data were compiled from at least three biological repeats.

Supplementary Figure 3 MTT assay of RAW264.7 macrophages treated with vehicle or PFG30 aggregates. Macrophages were treated with PFG30 at concentrations ranging from 450 ng/ml to 1 mg/ml for 48 h at 37°C with 5% CO_2_. Cells were then incubated with MTT for 4 h before addition of 10% SDS in PBS with 5 N HCl to dissolve formazan crystals overnight. Absorbances were read at 570 nm with a 630 nm background. Cell numbers were estimated based on a standard curve from at least 3 technical replicates each over triplicate experiments.

Supplementary Figure 4 L929 cellular uptake of C6-loaded PFG30 nanoparticles. **(A–C)** R Representative microscope images (magnification 10X) of PFG30 + C6 nanoparticle uptake by L929 mouse fibroblast cell line after 60 min incubation time. L929 cells were incubated at 37°C with PFG30 encapsulated C6 as reported in materials and methods section for RAW264.7 macrophages.
