# Supplementary figures and images for: Thermoresponsive Copolymer Nanovectors Improve the Bioavailability of Retrograde Inhibitors in the Treatment of Leishmania Infections
Source: Front Cell Infect Microbiol. 2021 Aug 19;11:702676. doi: 10.3389/fcimb.2021.702676 (PMC8417477; doi:10.3389/fcimb.2021.702676)

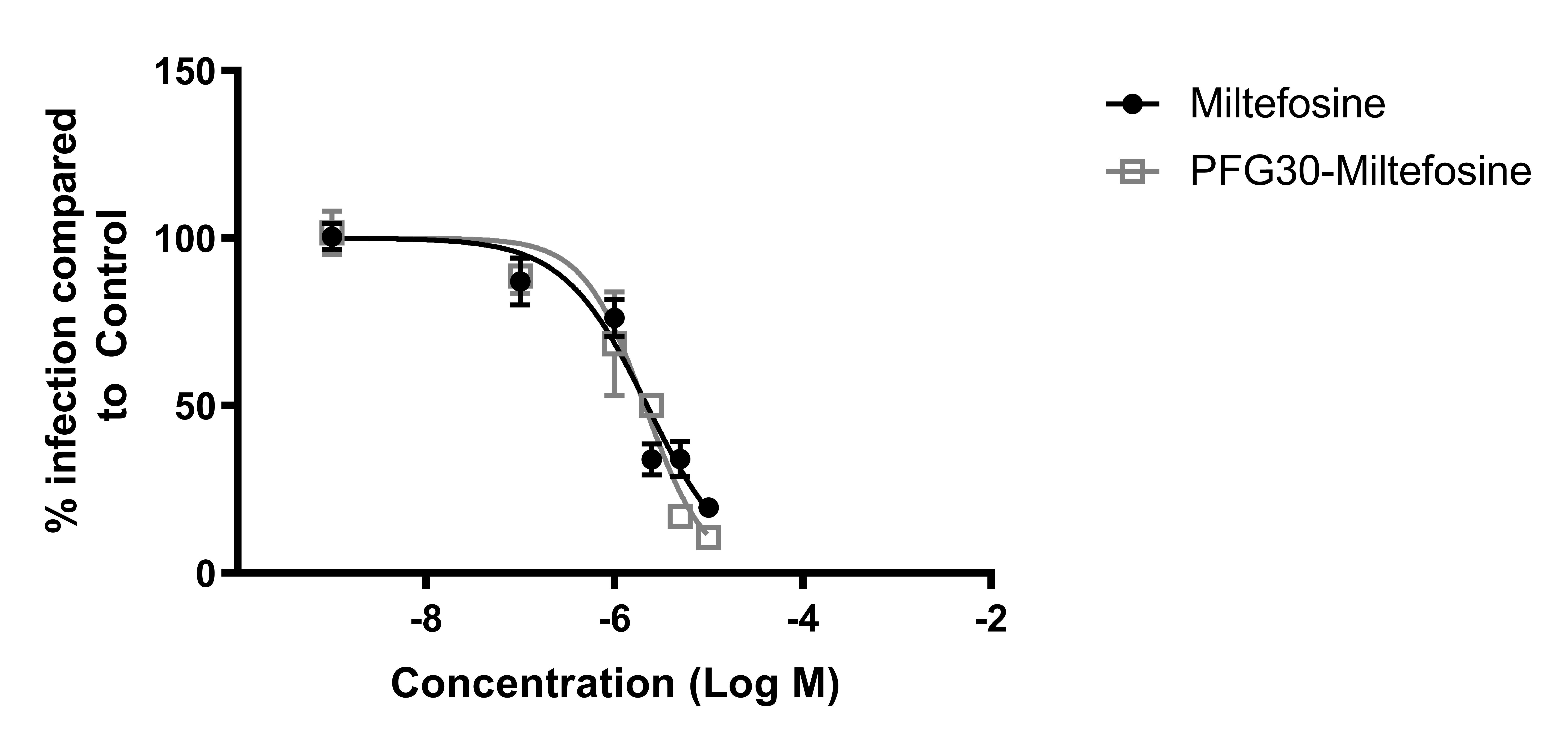

Supplement: Supplementary Figure 2 — Evaluation of polymer-encapsulated miltefosine on L. amazonensis-infected RAW264.7 macrophages. To L. amazonensis-infected RAW264.7 macrophages, free or PFG30 encapsulated miltefosine was added. After 48 h treatment, cells were fixed in 2% PFA in PBS. IFA’s were performed for detection of LAMP-1 and cell and parasite nuclei with DAPI. At least 200 cells were scored per coverslip and infection rates were standardized to a vehicle control before EC50 estimation. Data were compiled from at least three biological repeats. [file Image_2.tif]

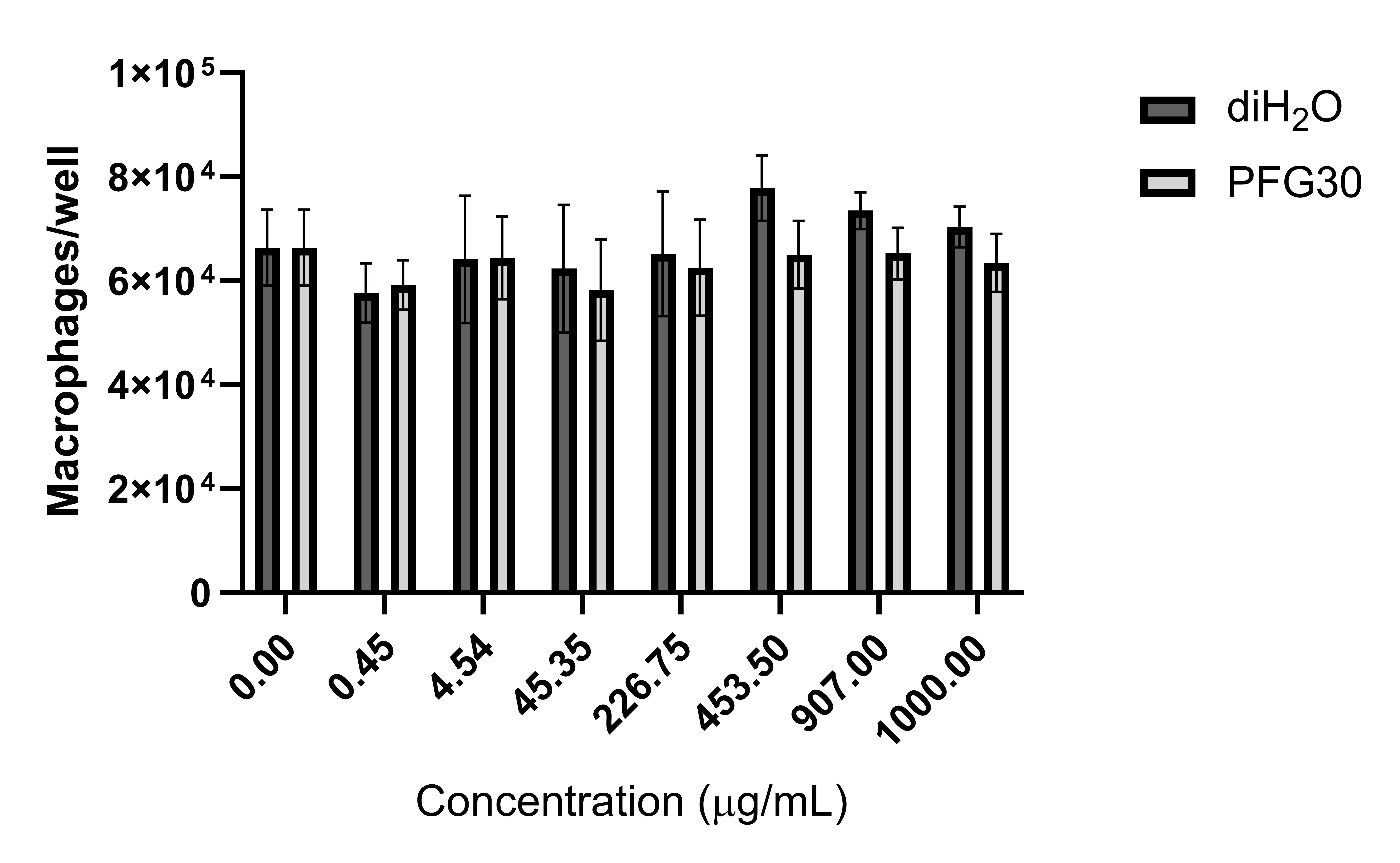

Supplement: Supplementary Figure 3 — MTT assay of RAW264.7 macrophages treated with vehicle or PFG30 aggregates. Macrophages were treated with PFG30 at concentrations ranging from 450 ng/ml to 1 mg/ml for 48 h at 37°C with 5% CO2. Cells were then incubated with MTT for 4 h before addition of 10% SDS in PBS with 5 N HCl to dissolve formazan crystals overnight. Absorbances were read at 570 nm with a 630 nm background. Cell numbers were estimated based on a standard curve from at least 3 technical replicates each over triplicate experiments. [file Image_3.tif]

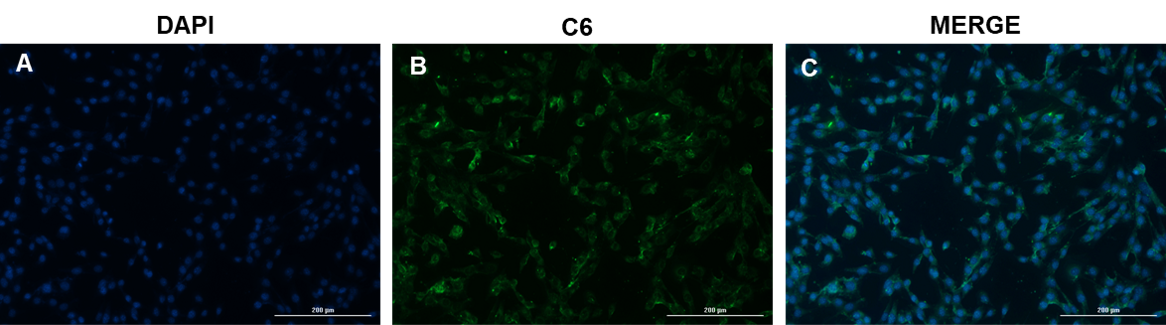

Supplement: Supplementary Figure 4 — L929 cellular uptake of C6-loaded PFG30 nanoparticles. (A–C) R Representative microscope images (magnification 10X) of PFG30 + C6 nanoparticle uptake by L929 mouse fibroblast cell line after 60 min incubation time. L929 cells were incubated at 37°C with PFG30 encapsulated C6 as reported in materials and methods section for RAW264.7 macrophages. [file Image_4.tif]
